# Mechanisms of Penetration Enhancement and Transport Utilizing Skin Keratine Liposomes for the Topical Delivery of Licochalcone A

**DOI:** 10.3390/molecules27082504

**Published:** 2022-04-13

**Authors:** Wenfeng Wu, Zhuxian Wang, Yufan Wu, Huiyi Wu, Tingting Chen, Yaqi Xue, Yuan Wang, Cuiping Jiang, Chunyan Shen, Li Liu, Hongxia Zhu, Qiang Liu

**Affiliations:** 1 School of Traditional Chinese Medicine, Southern Medical University, Guangzhou 510515, China; wenfengwu@hotmail.com (W.W.); wangzhuxian8998@163.com (Z.W.); yufanwu1118@163.com (Y.W.); chentingting0229@163.com (T.C.); xyqi1997@163.com (Y.X.); 19124335824@139.com (Y.W.); cuipingjiangcpu@163.com (C.J.); shenchunyan@smu.edu.cn (C.S.); shyshll@163.com (L.L.); 2 Department of Pharmacy and Nanfang Hospital, Southern Medical University, Guangzhou 510515, China; huiyi0214@yeah.net; 3 Integrated Hospital of Traditional Chinese Medicine, Southern Medical University, Guangzhou 510315, China

**Keywords:** topical drug delivery system, Licochalcone A, mechanisms of penetration enhancement, lipid nanoparticles

## Abstract

Keratin liposomes have emerged as a useful topical drug delivery system given theirenhanced ability to penetrate the skin, making them ideal as topical drug vehicles. However, the mechanisms of the drug penetration enhancement of keratin liposomes have not been clearly elucidated. Therefore, licochalcone A(LA)-loaded skin keratin liposomes (LALs) were prepared to investigate their mechanisms of penetration enhancement on the skin and inB16F10 cells. Skin deposition studies, differential scanning calorimetry (DSC), attenuated total reflection-Fourier Transform Infrared Spectroscopy (ATR-FTIR), and skin distribution and intracellular distribution studies were carried out to demonstrate the drug enhancement mechanisms of LALs. We found that the optimal application of LALs enhanced drug permeation via alterations in the components, structure, and thermodynamic properties of the stratum corneum (SC), that is, by enhancing the lipid fluidization, altering the skin keratin, and changing the thermodynamic properties of the SC. Moreover, hair follicles were the main penetration pathways for the LA delivery, which occurred in a time-dependent manner. In the B16F10 cells, the skin keratin liposomes effectively delivered LA into the cytoplasm without cytotoxicity. Thus, LAL nanoparticles are promising topical drug delivery systems for pharmaceutical and cosmetic applications.

## 1. Introduction

Licorice flavonoids are major components in *Glycyrrhizae Radix et Rhizoma*. More than 150 flavonoids and their derivatives have been isolated from licorice. Licochalcone A (LA) is a phenolic chalcone compound that is a species-specific component of *Licorice inflata*. Clinical and pharmacological studies have shown that LA possesses various features, including anti-inflammatory [1], anti-oxidant [2], anti-tumor [3], and immune-enhancing properties [4,5]. Moreover, it exerts a remarkable whitening effect by inhibiting melanogenesis and tyrosinase activity in B16 cells and ultraviolet radiation b (UVB)-exposed C57BL/6 mice, as we previously found [6,7,8]. However, it is poorly soluble in water and difficult to permeate into the skin, leading to inadequate skin concentrations. As such, it is necessary to facilitate LA transport into the skin using some effective methods, such as encapsulation with nano vehicles [6].

Topical administration is the second most important means of administering medication in addition to oral administration [7,9]. Not all drugs are applicable trans dermally due to the limitations of the skin barrier, which restricts the absorption of drugs. Poor drug absorption leads to low bioavailability and reduced overall efficacy. Ceramide is an important structural component in lipids [10]. It maintains normal skin-barrier function and also acts as a secondary messenger on the keratinocyte membrane, thus promoting keratinocyte proliferation and differentiation. At the same time, it performs physiological functions, such as moisturizing, adhesion [11], maintaining the skin barrier [12], anti-aging [13], anti-inflammatory [14], and anti-sensitivity functions, whitening, and apoptosis induction. Therefore, it is a useful and semi-bionic strategy through which to deliver active compounds into the skin using ceramide encapsulation.

Skin keratin liposomes are a new type of topical drug delivery system based on existing liposome technology. Zuo prepared cryptotanshinone-loaded keratin liposomes for topical use and improved drug’s bioavailability in the skin and its anti-acne effect [15]. Yang established indocyanine-green- and L-menthol-loaded hybrid cerasomes as a multifunctional theranostic nanoplatform for US imaging and photothermal therapy [16]. This method utilizes ceramides, cholesterol, palmitic acid, and cholesterol sulfate. These compositions are similar to the stratum corneum. Skin keratin liposomes can significantly improve the skin penetration of drugs due to their high affinity and compatibility with keratin, as well as enhancing drug stability and targeting the hair follicles. In addition, skin keratin lipids do not require the use of surfactants and co-surfactants, which could reduce skin irritation upon exposure to preparations [17]. Thus, skin keratin liposomes are highly effective, leading to fewer side effects when encapsulating drugs. However, the mechanisms through which skin keratin liposomes improve drug penetration into the skin have not been explored elsewhere.

Therefore, in the present work, we report for the first time the preparation of LA-loaded skin keratin liposomes (LAL) using skin keratin lipids to enhance LA retention, and then characterize the underlying penetration enhancement mechanisms of skin keratin liposomes. The encapsulation efficiency, zeta potential, poly dispersity index (PdI), and particle sizes of the LALs were investigated to obtain the optimal formulation. Next, skin deposition studies and in vitro release experiments were performed to reveal the transdermal properties of the LALs. Subsequently, differential scanning calorimetry (DSC), attenuated total reflection–Fourier transform infrared spectroscopy (ATR-FTIR), and skin distribution and intracellular distribution studies were carried out to demonstrate the penetration enhancement mechanisms of the LALs. Finally, cytotoxicity experiments and a skin irritation study were conducted to explore the formulation’s safety when applied on the skin.

## 2. Results and Discussion

### 2.1. LA Skin Keratin Liposome (LAL) Characteristics

**Figure 1:**
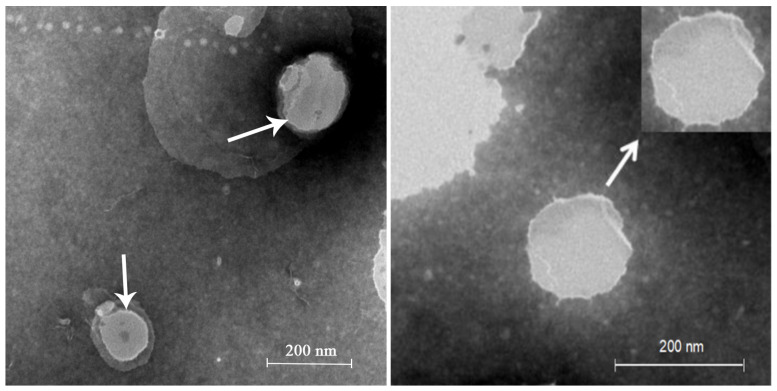
TEM micrographs of LAL nanoparticles.

The optimal LAL preparations were achieved using 25.0 mg soya lecithin, 25.0 mg cholesterol, 0.3 mg LA, 0.025 mg ceramide, 3 mL ethanol, and 10 mL phosphate buffer (pH 7.0), which had been preheated to 65 °C. The encapsulation efficacy (EE) percentage of the LALs was 54.07 ± 6.99%.Moreover, the LALs possessed an average particle size of 186.7 ± 1.31 nm and a zeta potential of −2.54 ± 0.19 mV, with a PDI value of 0.283 ± 1.31. The morphology of the LALs is shown in Figure 1, which demonstrates that LAL liposomes were spherical. It can be observed that the particle sizes of the LALs were approximately 180 nm, which was similar to those determined through dynamic light scattering (DLS). Both the mean particle size and the PDI are important features of liposomes, from which the stability of the drug-loaded nanoparticles can be predicted [18,19]. The distribution of nanoparticles can be influenced by the particle size, and a small particle size usually leads to a narrow PDI and vice versa. Although the measurement principles of the transmission electron microscopy (TEM) and DLS were distinct, the results ofthe size distribution were similar, indicating the stable properties of the LAL formulations.

### 2.2. Skin Retention and Dialysis Bag Experiments

**Figure 2:**
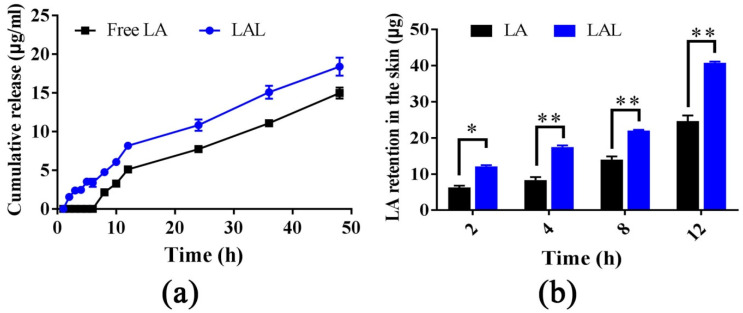
(**a**) The release profile of LA-loaded skin keratin liposomes over48 h (*n* = 3); (**b**) the skin retention of LAL nanoparticles and free LA at 2, 4, 8, and 12 h (* *p* < 0.05, ** *p* < 0.01 vs. LA, *n* = 3).

Franz diffusion cells were used to investigate the permeation and the retention properties of the free LA and the LAL. Moreover, the dialysis bag method was applied to determine the LA release from encapsulated liposomes. The dialysis bag experiments revealed that the LALs had an initial slow-release phase in the first 6 h followed by a faster release phase that lasted until the end of the 48 h (Figure 2a). Moreover, the LALs displayed a higher release amount and release rate than the free LA at all the time points; however, there no significant difference was observed. The release rates of the LALs and free LA were 3.28 μg/h/cm^2^ and 2.67 μg/h/cm^2^, respectively. However, the results were the opposite of previous results [9,20,21], which demonstrated that the release rate and release amount of the free drug was higher than the nanoparticle-derived drugs. This was possibly due to the low solubility of LA in the release medium(30% PEG400/70% saline) and the easier disintegration of the LAL nanoparticles, resulting in the higher release capability of the LAL compared with the free LA. Furthermore, the free LA was not able to permeate the rat skin; therefore, it could not be detected into the receptor phase, even when increasing its concentration, due to it slow water solubility and high log P value. In addition, the skin keratin liposomes could not facilitate LA permeation through the skin. However, both free LA and LALs were detected in the skin retention studies (Figure 2b).

The log Pof the LA was 4.95.Therefore, the LA could not permeate through the rat skin in its free form [7]. This demonstrated that the liposolubility of the LA resulted in inevitably poor skin diffusion ability. As shown in Figure 2b, the retention of the LA in the skin treated by the LALs was significantly higher than that of the LA at four time points. At 12 h, the LA retention in the rat skin was 24.380 μg, and it was 39.335 μg for the free LA and the LALs, respectively. These findings suggested that the skin keratin liposome system could promote the retention of LA in the skin. This was probably because the ceramide exhibited high affinity with keratin in the skin, decreased the trans epidermal water loss, and increased the hydration of the skin [22], thereby leading to a higher level of retention.

### 2.3. Attenuated Total Reflection-Fourier Transform Infrared Spectroscopy (ATR-FTIR) Test of SC Components

**Figure 3:**
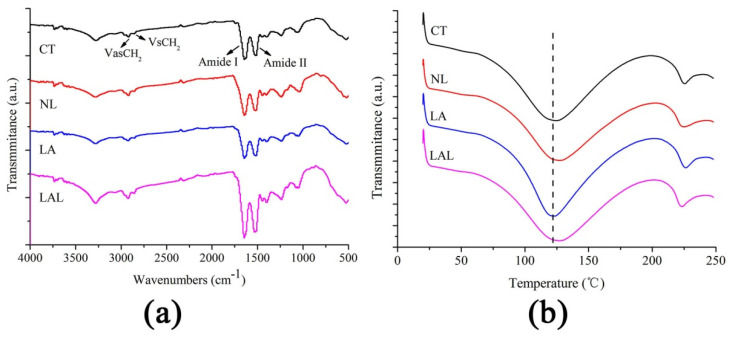
(**a**) FTIR spectra of skin samples treated with different formulations; (**b**) the DSC thermograms of skin samples after treatment with LALs and other formulations.

Figure 3a and Table 1 depict the findings of the FTIR analysis. The peaks at 2900–2800 cm^−1^ represent the VasCH_2_ and VsCH_2_ of the SC lipids, while the peaks at 1600–1500 cm^−1^ are the Amide I and Amide II of the keratin [21,22,23]. The VasCH_2_ and VsCH_2_ peak moved to 2928.43 cm^−1^ and 2859.27 cm^−1^, respectively, after treatment with LALs, when compared to the control group. The results demonstrated that LALs could disrupt the lipid order to improve penetration. Moreover, the blank-skin keratin liposomes (NL) and LA also induced the movement of the VasCH_2_ and VsCH_2_ peak, but the movement displacement was lower than that of the LALs. For the Amide I and Amide II peaks, the NL, LA, and LALs all led to different movements. This suggested that the NL and LALs could transform the protein structure to promote the drug penetration. In addition, the peak absorption area of the groups exposed to the LALs and NL were significantly reduced compared with those of the control group. That of the LAL group was the lowest.

The FTIR analysis revealed the underlying mechanism of LA penetration improvement by skin keratin liposomes based on the change in SC structure. The reduced peak areas and peak height of the Amide I and Amide II suggested that the keratin structures were partly transformed into β-folding shapes from α-helical shapes after treatment with LALs [23,24,25]. Additionally, the keratin and lipid peak areas were drastically lower than those of the control group, indicating that the permeability of the skin keratin lipids was dependent on the extraction of keratin and SC lipids [26,27]. Altogether, enhancing lipid fluidization and altering skin keratin might be the mechanisms behind the enhanced drug penetration of skin keratin liposomes.

### 2.4. Differential Scanning Calorimetry (DSC) Study of SC Thermotropic Properties

DSC was used to investigate the alteration in the thermodynamic properties of the SC treated by LALs and other formulations. Keratin is a temperature-sensitive compound that changes its protein structure upon exposure to external thermal energy. Figure 3b demonstrates the DSC curves of the skin samples. The characteristic keratin denaturation peaks are seen at 110–120 °C [28]. Table 1 shows that the LAL keratin melting points were remarkably reduced in comparison to the control group. A more dramatic reduction was noted in the NL group. However, the keratin melting point of the CT groups was lower than that of the free LA groups. The enthalpy of the differently treated groups (Table 1) increased to a different degree compared with the control, especially the LAL > NL > LA groups.

Treated with NL and LAL, the phase transition temperature of keratin decreased, indicating that LALs can effectively deliver LA into the skin [29]. In addition, the decreased melting temperature and increased enthalpy of the skin treated with LAL formulations reflected the changed keratin structure, indicating that skin keratin lipids could reduce the SC barrier’s function by changing the helix structure of keratin [30]. Altogether, LALs can alter lipid order and keratin structure for enhanced drug penetration.

### 2.5. Skin Distribution of Nanoparticles

**Figure 4:**
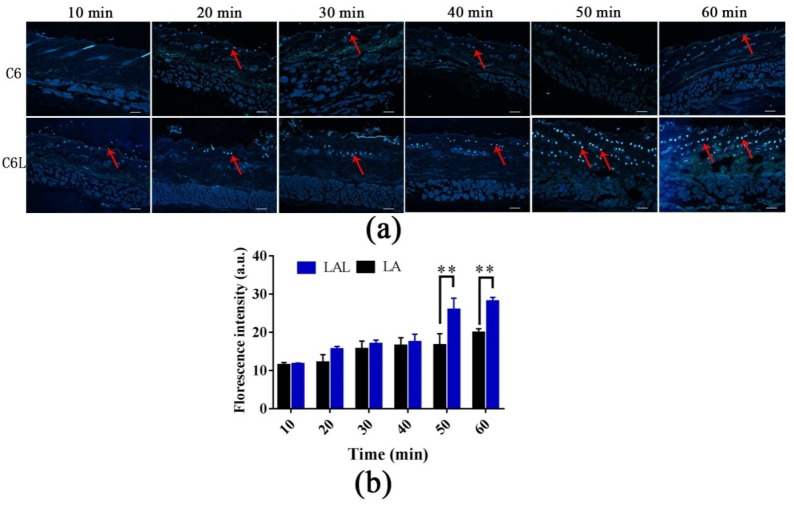
(**a**) The fluorescence images of the skin samples treated with C6 and C6L for 10, 20, 30, 40, 50, and 60 min. The scale bar is 100 μm; (**b**) the quantitative florescence intensities of C6 and C6L into skin at different time (** *p* < 0.01 vs. C6, *n* = 3).

In this part, C6 was used to as a substitute for LA to prepare C6-loaded skin keratin liposomes (C6L) [31]. As shown in Figure 4a, the fluorescence signal in the C6L groups was first visualized in the hair follicles before it was distributed uniformly in the skin after 1 h. The fluorescence intensities and penetration rates in the C6L-treated skin were all significantly higher than those of the C6 group at all time points. After 1 h, C6 delivered by skin keratin liposomes can permeate into the entire hair follicle and deposit at the follicle’s root. The result demonstrated that hair follicles were the main penetration pathways of the C6L.

Moreover, the C6L penetrated deeply into the skin in a time-dependent manner (Figure 4b). The fluorescence intensities of the C6L into the skin at 50 min and 60 min were significantly higher than those of the free C6. The LA fluorescence mainly accumulated into the hair follicles at different times [30]. This indicated that the hair follicle served as a drug reservoir for sustained drug release. These findings were consistent with the results of the in vitro penetration [32]. This further demonstrated that skin keratin liposomes are effective vehicles for LA delivery into the deeper skin layers through hair-follicle pathways.

### 2.6. Cytotoxicity Experiments

**Figure 5:**
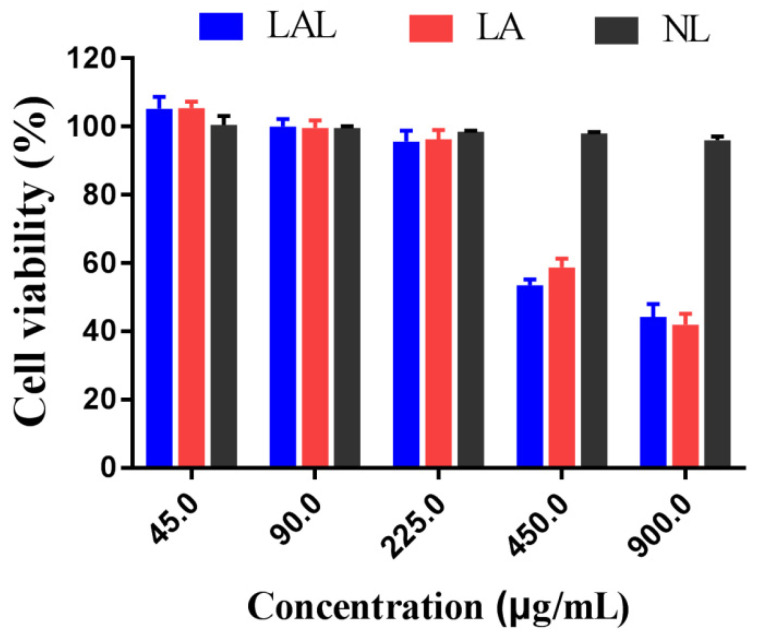
B16F10 cell viability after treatment with LAL, LF, and NL formulations for 24 h (*n* = 6).

The NL had more than a 90% cell survival rate at different concentrations (Figure 5), a finding that is consistent with previous reports demonstrating that lipid nanoparticles possessed compatible non-ionic surfactants and glycerides that were safer to B16F10 cells [33]. Moreover, the B16F10 cell viability was positively correlated with the LAL concentration, which was lower than 85% at 225 μg/mL. These results demonstrated that the LAL formulation was safe for the B16F10 cells when the concentrations were below 225 μg/mL.

### 2.7. Intracellular Distribution of LAL Nanoparticles

**Figure 6:**
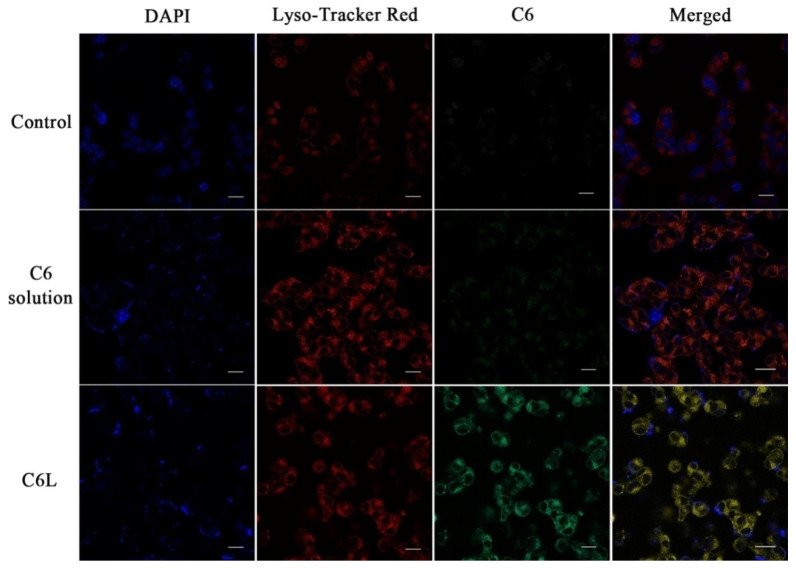
Intracellular distribution of free C6 and C6L micelles in B16F10 cells (scale bar: 10 μm).

Figure 6 demonstrates B16F cell nuclei (blue), C6 (green), and lysosome (red) in the B16F10 cells. We can see that the C6L groups demonstrated stronger C6 fluorescence, in contrast to the C6 group. The yellow signals indicated a mix of green (C6) and red (lysosome) signals. The co-localization results of the studies suggested that there was a reduced P-glycol protein efflux and increased cytoplasmic uptake in the C6L groups [34,35]. Moreover, C6Lnanoparticles penetrated into the cells via the endosomal–lysosomal pathway. Nanoparticle-containing drugs are easily destroyed by endosomal and lysosomal enzymes in acidic conditions [23,36]. The luminance change in the Lyso-Tracker Red in the C6L groups indicated that the vesicles ruptured to release the carriers.

### 2.8. Skin Irritation

**Figure 7:**
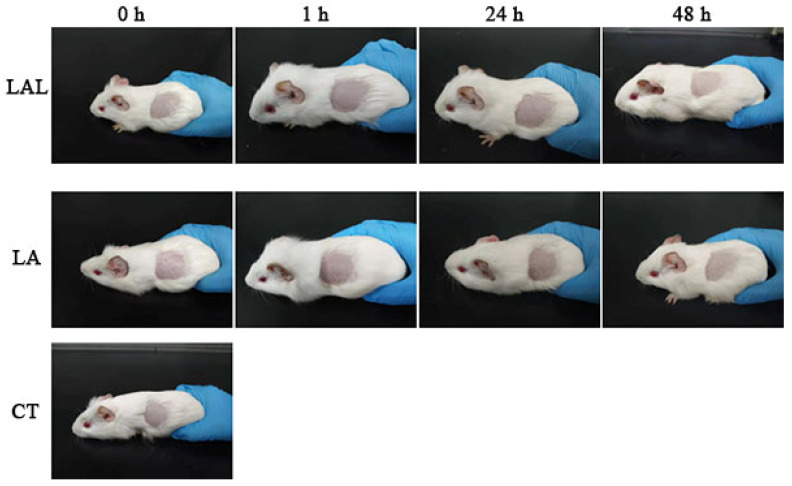
Photograph of the abdomens of guinea pigs exposed to the various formulations.

Figure 7 shows the skin irritation on the guinea pig skins treated by different formulations. The degree of skin irritation was observed at 1, 24, and 48 h after exposure to normal saline, NL, or LAL. There was no irritation on the treated groups, which indicated that the NL and LALs did not cause any skin irritation, suggesting that LAL was a safe topical drug delivery system.

## 3. Materials and Methods

### 3.1. Materials

LA was procured from Chengdu Luokema Biological Technology Co. (Chengdu, China). Cholesterol and soya lecithin were obtained from Shanghai Mecklin Biological Technology Co. (Shanghai, China). Polyethylene glycogen I 400 (PEG400) was obtained from Beijing Solabor Biological Technology Co. (Beijing, China). Lyso-Tracker Red, ceramide, 2-(4-Amidinophenyl)-6-indolecarbamidine dihydrochloride (DAPI), Cell Counting Kit-8 (CCK-8), trypsin, fetal bovine serum (FBS), and Dulbecco’s modified Eagle’s medium (DMEM) were bought from KeyGEN Bio TECH (Jiangsu, China). Sigma-Aldrich (St. Louis, MO, USA) supplied Coumarin-6. All other reagents used in this experiment were of analytical quality.

The Southern Medical University provided male Sprague–Dawley (SD) rats (200 ± 10 g). All animal experiments performed conformed to the animal handling guidelines established by the “Guiding Principles in the Care and Use of Animals”, and the procedures were approved by the Ethics Committee of Southern Medical University (L2019036, date of approval: 13 April 2019).

### 3.2. Quantification of LA

HPLC (Agilent 1260) was used to quantify LA concentrations (Agilent 1260 series, USA). The flow rate of the mobile phase comprised acetonitrile–methanol–water with 0.2% phosphoric acid (15/10/75, *v*/*v*/*v*) at 1 mL/min, with a detection wavelength calibrated at 372 nm.

### 3.3. Optimizing the Preparation Condition of LAL

#### 3.3.1. Preparation of LAL

Solution A: a total of 25.0 mg soya lecithin, 25.0 mg cholesterol, and 0.3 mg LA was dissolved into 3 mL ethanol. Solution B: 25.0 mg ceramide was mixed with 10 mL phosphate buffer (pH 7.0), which had been preheated. Next, solution A was steadily mixed into solution B. The mixed solution was kept under agitation at 750 rpm at room temperature for 20 min. Next, a water bath was used to incubate the solution for 50 min at 65 °C, followed by filtering using a 0.45-micrometer microporous membrane. The final product was stored at 4 °C until analysis. As a control, blank-skin keratin liposomes were prepared with the same methods.

#### 3.3.2. Encapsulation Efficiency of the LALs

The encapsulation efficiency was estimated using the ultracentrifugation method. LAL formulations were collected after centrifugation at 16,000 rpm for 45 min. The supernatant was discarded, followed by the addition of 1 mL methanol to completely dissolve the precipitate. EE of the nanoparticles was analyzed by HPLC and calculated using Equation (1).

EE (%) = W_Enca_/W_Total_ × 100% (1)

#### 3.3.3. The Zeta Potential, Poly Dispersity Index (PdI), and Particle Size

LALs were analyzed using the Mastersizer particle size analyzer 2000 and Malvern Zetasizer Nano ZS (Malvern, Worcestershire, UK). The nanoparticles were negatively stained with 3% aqueous solution of phosphotungstic acid and dried on a microscopic carbon-coated grid for 30 min. The morphologies of all preparations were then visualized and photographed on a TEM (HITACHI, H-7650) with a voltage of 80 kV.

### 3.4. In Vitro Permeation Studies of LALs

#### 3.4.1. Skin Deposition Studies

An in vitro skin permeation experiment was used to determine the amount of LA retained in the abdominal skin of the SD rats. The SD rats were anesthetized by using an intraperitoneal injection of 3% sodium pentobarbital and had their abdominal fur removed using depilatory paste. Next, the subcutaneous fatty tissue on the abdominal skin was carefully removed, and the final skin was obtained. The skin was then sandwiched between the donor and receptor compartments of Franz diffusion cells with the dermis side facing forward. Phosphate buffer solution (PBS) (pH: 7.4)/PEG400 (*v*/*v* 4:1) served as the release medium, which was stirred at 37 °Cand 350 rpm. Selected time points were 2, 4, 8, 12, and 24 h. PBS solution was used to rinse the skin surfaces (both sides) to remove donor cells. Skin was cut into pieces and extracted in ethanol–acetonitrile (1:1, *v*/*v*) using ultrasound methods at 100 W for 1 h. The mixture was centrifuged for 30 min at 10,000 rpm and filtered by 0.22-micrometer microporous membrane. HPLC was then used to evaluate LA concentrations.

#### 3.4.2. In Vitro Dialysis Experiments

A dialysis bag method was used to investigate in vitro release. A 1-milliliter LAL solution was poured into a dialysis bag, and the entire bag was immersed for 48 h at room temperature. The dissolve medium was 100 mL 30% PEG400/70% saline (*v*/*v*). A total of 1 mL of dissolve medium was withdrawn from the sampling port at 0.5, 1, 2, 3, 4, 5, 6, 8, 10, 12, 24, and 48 h. After withdrawing the sample, the medium was immediately replenished with an equal volume of fresh receptor phase. HPLC was used to analyze the drawn sample to assess the cumulative release (%) of LA.

#### 3.4.3. Differential Scanning Calorimetry (DSC)

The treated skin samples were processed for skin deposition studies. After the permeation studies, the skins were cleaned with saline and lyophilized. The lyophilized skin samples were cut into pieces and placed in the aluminum DSC pans of the differential scanning calorimeter (TA Q2000America TA, New Castle, Lindon, UT, USA) to investigate the effects of normal saline, LALs, and NL on SC lipids and keratin of the skin. Nitrogen was used as the gas flow. The temperatures used were between 20 and 250 °C with a heating rate of 10 °C/min.

#### 3.4.4. Fourier Transform Infrared Spectroscopy (FTIR)

The treated skin samples were also processed for skin deposition studies. After the permeation studies, the skins were cleaned with saline and lyophilized. The lyophilized skins were directly placed into FTIR spectrophotometer (Nicolet iS50 FT-IR spectrometer, American Thermos, New York, NY, USA) to investigate the SC structure alterations in skin samples. For the FTIR analysis, the samples were analyzed at a resolution of 2 cm^−1^ and a wavenumber range of 500 to 4000 cm^−1^.

#### 3.4.5. Skin Distribution Analysis

Fluorescence microscopy was applied to visualize LALs’ dynamic transport behaviors across the skin. LA itself emits green fluorescence, thus negating the use of additional reagents. Here, skin samples were exposed to LALs, LA solution, and control reagents using Franz diffusion cells for 10, 20, 30, 40, 50, and 60 min. Samples were then fixed with 4% paraformaldehyde and treated with confocal laser scanning microscopy (CLSM) to observe the distribution of LA solution and LALs in skin.

#### 3.4.6. Intracellular Distribution and Cellular Uptake of Lipomes

LALs’ intracellular distribution behaviors were further investigated on B16F10 cells. B16F10 cells were cultured with DMEM supplemented with 10% fetal bovine serum (FBS) at 37 °C in 5% CO_2_. A fluorescence microscope was used to obtain images of cells incubated with different treatments. B16 cells were allowed to culture on creep plates before being exposed to LA and LALs for 8 h. The cultured cell lysosomes were then stained for another 30 min with Ly so-Tracker Red. Samples were then rinsed with PBS (pH = 7.4) and fixed with aqueous paraformaldehyde solution (4%, *w*/*v*) for 15 min at room temperature. The samples were then stained with DAPI to visualize the cell nuclei.

### 3.5. In Vitro Cytotoxicity Experiments

Cells were cultured in an incubator with 37 °C saturated humidity at 5% carbon dioxide atmospheric concentration. Cells were collected and plated onto a 96-well plate at a density of 2 × 10^4^ cells and 100 µL/well. The cells were cultured for 24 h before the addition of 100 μL LA or LAL solution. This mixture was then allowed to incubate for another 24 h. Later, the cell plates were taken out of the incubator and 10 μL of the CCK8 solution was added to each well, respectively, and incubated for 30 min. Finally, cell proliferation was quantified using an absorbance measurement at 450 nm.

### 3.6. Skin Irritation

In this study, six guinea pigs were randomly allocated into each of the treatment groups. The backs of the guinea pigs were shaved and depilated with a depilatory cream. The guinea pigs were exposed to various formulations (normal saline, NL, and LAL) for 48 h to investigate the degree of skin irritation. The formulations were cleaned after 48 h with normal saline. Next, the degree of skin irritation (Table 2) after the application of the different formulations at 1, 24, and 48 h was observed. The occurred cerate (%) was calculated as follows: Occurring rate (%) = Number of irritated animals/Total number of animals × 100%.

### 3.7. Data Analysis

All data were described in terms of X ± D. Data analysis was carried out using the SPSS software version 17.0, with both the T-test and least-significant difference (LSD) methods used. A *p*-value of <0.05 was used to indicate statistical significance.

## 4. Conclusions

In the present study, LA was successfully encapsulated by skin keratin liposomes to formulate LALs for topical delivery with no skin irritation. The LALs proved to be optimal in terms of their particle size, zeta potential, and drug encapsulation. The skin keratin liposomes enhanced the LA skin delivery through the hair follicles by enhancing the lipid fluidization, altering the skin keratin, and changing the thermodynamic properties of the SC. Moreover, the skin keratin liposomes could also effectively deliver C6 into the B16F10 cells. These were the potential mechanisms behind the enhanced drug penetration by the skin keratin liposome vehicles, which have great potential for use in the pharmaceutical filed.

## Figures and Tables

**Table 1:** Peaks of SC lipids and keratin of skin samples after treatment with different formulations.

Sample	Lipids		Keratin		Keratin	
	VasCH_2_	VsCH_2_	Amide I	Amide II	Melting Temperature (°C)	Enthaly (J/g)
LA	2923.12	2856.22	1585.93	1531.20	120.71	322.13
LAL	2928.43	2859.27	1584.04	1535.92	115.24	351.60
NL	2925.68	2856.14	1577.66	1542.62	111.65	336.78
CT	2922.36	2858.31	1588.15	1518.49	118.96	335.39

**Table 2:** Skin irritation.

Occurrence Rate (%)	Level	Strength	Skin Irritation Definition
0–8	I	None	No erythema and no hydroderma
9–28	II	Mild	Mild erythema and mild hydroderma (barely visible)
29–64	III	Moderate	Moderate erythema and moderate hydroderma (obviously raised)
65–80	IV	Severe	Severe erythema and severe hydroderma (the skin humps 1 mm with the outline clear)
81–100	V	Extreme	Purplish red erythema or mild eschar hydroderma, extreme hydroderma (skin humps of more than 1 mm with the outline expansile)

## Data Availability

Not applicable

## Sample Availability

Not available.
